# Effective media communication of disasters: Pressing problems and recommendations

**DOI:** 10.1186/1471-2458-7-97

**Published:** 2007-06-06

**Authors:** Wilson Lowrey, William Evans, Karla K Gower, Jennifer A Robinson, Peter M Ginter, Lisa C McCormick, Maziar Abdolrasulnia

**Affiliations:** 1 Department of Journalism, University of Alabama, Tuscaloosa, USA; 2 Department of Telecommunication and Film, University of Alabama, Tuscaloosa, USA; 3 Department of Advertising and Public Relations, University of Alabama, Tuscaloosa, USA; 4 Department of Public Relations, University of Florida, Gainesville, USA; 5 Department of Health Care Organization and Policy, University of Alabama at Birmingham, Birmingham, USA; 6 South Central Center for Public Health Preparedness, University of Alabama at Birmingham, Birmingham, USA

## Abstract

**Background:**

Public health officials and journalists play a crucial role in disseminating information regarding natural disasters, terrorism and other human-initiated disasters. However, research suggests that journalists are unprepared to cover terrorism and many types of natural disasters, in part because of lack sufficient expertise in science and medicine and training. The objective of this research was to identify solutions to problems facing journalists and public health public information officer (PIOs) of communicating with the public during natural and human-initiated disasters.

**Methods:**

To assist in identifying the most pressing problems regarding media response to health-related risks such as terrorism and large-scale natural disasters, 26 expert advisors were convened, including leaders representing journalists and public information officers, state health officials, experts in terrorism and emergency preparedness, and experts in health, risk, and science communication. The advisory group participated in pre-arranged interviews and were asked to identify and review bioterrorism educational resources provided to journalist. All advisory group members were then invited to attend a day long meeting January 29, 2004 to review the findings and reach consensus.

**Results:**

The most pressing problems were found to be a lack of coordination between PIO's and journalists, lack of resources for appropriately evaluating information and disseminating it efficiently, and a difference in perception of PIO's and journalist towards each others role during emergency situations. The advisory board developed a list of 15 recommendations that may enhance communication plans betweens PIO's, journalist and the public. The solutions were meant to be feasible in terms of costs and practical in terms of the professional and organizational realities in which journalists and PIO's work.

**Conclusion:**

It is clear that PIO's and journalists play crucial roles in shaping public response to terrorism and other disasters. The findings from this formative research suggest that perspectives and organizational processes often limit effective communication between these groups; though practical solutions such as participation of journalists in drills, scenario exercises, sharing of informational resources, and raising awareness at professional trade meetings may enhance the timely dissemination of accurate and appropriate information.

## Background

There has been a dramatic increase in the number of natural and human initiated disasters. From 1900 to 2006 there have been 15,833 reported natural and human initiated disasters worldwide. One third of all these disasters have occurred between 2000 and 2006 [1]. Many factors are contributing to this increase including population growth, urbanization, increasingly complex technologies, a rise in world-wide terrorism, social unrest, global economic and social interdependence, the emergence and re-emergence of infectious diseases such as cholera, yellow fever, diphtheria, malaria, and plague and the emergence of new infectious diseases including HIV, Ebola, Hepatitis C, hantavirus, and rotavirus.

Clearly, journalists and news organizations play crucial roles in disseminating information regarding natural disasters, terrorism and other human-initiated disasters [2]. For example, newscasts were the primary source of information for people trapped after hurricanes Katrina and Rita slammed into to the US Gulf Coast. Similarly, the media informed the world of the massive destruction, loss of life, and need for international aid after the 2004 tsunami which killed over 300,000 and left millions homeless in South Asia, India, Sri Lanka, Africa, Thailand, and Indonesia.

Most adults in the United States first learned of the September 11, 2001 attacks via mass media. Television was the primary source of this news, although audiences also relied on radio, newspapers, newsmagazines, and online media [3-5]. The bioterrorism-related anthrax outbreak in fall 2001 generated immense media attention. The emergence of SARS in 2003 was also covered frequently and prominently in news outlets around the world. Further, the media played a vital role in informing the public about the sources and risks of terrorism reporting in 2004 on the four commuter trains that were bombed during morning rush hour in Madrid, Spain killing 191 people and injuring 1,200 others and in July 2005's coordinated attack on London's underground transportation system which left 56 dead and 700 injured.

News reports of terrorism and natural disasters, however, sometimes have been faulted for inaccurate, incomplete, and sensational coverage that may contribute to public misunderstanding of risks [6-8]. Research suggests journalists are unprepared to cover terrorism and many types of natural disasters, in part because journalists lack sufficient expertise in science and medicine [9-11].

Responding to these concerns and acknowledging that public health is playing an increasingly important role in disaster response and communication, The Centers for Disease Control and Prevention (CDC) has launched several ambitious training programs to improve the ability of public health professionals to communicate through journalists about terrorism and disaster preparedness. In coordination with the Association of Schools of Public Health, the CDC supports 27 Centers for Public Health Preparedness that are located within accredited schools of public health. The project discussed in this paper is a product of one such partnership, the South Central Center for Public Health Preparedness, which is based in the Schools of Public Health at the University of Alabama at Birmingham and Tulane University.

These training programs have been informative and past studies on journalists' decision-making shed light on the problem. Yet there is still no consensus regarding the most pressing challenges facing journalists and public health public information officer (PIOs) as they respond to natural and human-initiated disasters. This paper identifies the key challenges and recommends possible solutions.

### Public health's expanding role in preparedness

Historically, public health's response to disasters has been improvised and impromptu. Health departments were called upon in response and recovery operations as an afterthought, leaving the medical and health efforts related to emergencies uncoordinated with the rest of the emergency response. However, since September 11, 2001 there has been a new emphasis on disaster and emergency preparedness in public health. Further, there has been a concerted effort to integrate disaster and emergency preparedness into traditional public health functions and organizational structures. Today, in most communities, there is mainstream recognition that public health is a key player in emergency response.

The public health system is a complex network of individuals and organizations that assure conditions in which people can be healthy in the community. The "public health system" is traditionally thought of as being made up of local and state health departments and laboratories, the health care delivery system, and the public health and health sciences divisions of academia. In order to protect the public's health during crises this network must expand to include traditional response agencies such as emergency management agencies, fire department, law enforcement, and other entities with a responsibility to protect the safety and health of the public. Other volunteer and faith-based organizations must be included so that their resources can be utilized to the fullest extent during response and recovery operations. Another important partner in this network is the media. Public health officials realize that the media can shape public opinion and influence decision making that can influence population health and welfare. The media can serve as a major outlet to provide information that the public needs during an emergency on personal protective actions and strategies. In 2003, the Institute of Medicine, in its report *The Future of Public Health in the 21*^st^*Century*, examined the lack of understanding that editors and journalists and medical and public health officials typically have of each other's perspectives or objectives. A clear need was identified in both the journalism and public health communities for training, research, and dialogue to improve this relationship and their abilities to accurately inform and communicate with the public during any public health emergency [12].

### Professional and organizational realities

Studies show that to improve journalists' expertise it is necessary to deal with the organizational and professional factors that constrain news coverage. For example, because news events are unpredictable, news managers encourage reporters to be generalists rather than specialists [13]. In addition, turnover rates are relatively high among journalists, who typically advance in their careers by moving from one news outlet to another. As a result, relatively few journalists stay in one community long enough to become well acquainted with its public health institutions. Because journalists typically work quickly and under deadlines, they find it difficult to produce rigorous, in-depth reports.

Journalists see themselves in multiple professional roles. For example, they are both channels of information and "watchdogs" on government institutions [14]. Journalists aim to inform the public about health risks, but they feel obligated to go beyond passive dissemination of information. Journalists may adopt a wary and even skeptical stance regarding government health agencies and spokespersons.

For their part, public health agencies have been faulted for not preparing in advance information useful in emergencies and for ineffective dissemination of information [15]. Some critics even contend that government health agencies and their PIOs frequently obstruct rather than facilitate communication [16,17]. But PIOs are also hemmed in by organizational factors. They have the joint burden of improving understanding between content experts and journalists, and ensuring that complex information about health is communicated accurately [18]. PIOs are also constrained by legal requirements designed to protect citizen privacy.

## Methods

To assist in identifying the most pressing problems regarding media response to health-related risks such as terrorism and large-scale natural disasters, 26 expert advisors were convened, including leaders representing journalists and public information officers, state health officials, experts in terrorism and emergency preparedness, and experts in health, risk, and science communication. The advisory group members were interviewed via [3] prearranged conference calls, [4] prearranged telephone calls with individual advisors, and [5] e-mail correspondence. The advisors were asked to identify what they believed to be the most pressing problems. They were also asked to respond to concerns raised in the emerging literature regarding news coverage of terrorism and other disasters. The advisors evaluated resources developed to support journalists, such as *A Journalist's Guide to Covering Bioterrorism *[14] and were invited to nominate resources they have found helpful. These conversations began in summer 2003 and culminated in a daylong meeting on January 29, 2004, on the campus of the University of Alabama at Birmingham. The meeting was attended by 20 of the 26 advisors. During this meeting the advisory group reviewed and discussed reports in which summarized pre-meeting interviews. Table 1 presents the advisory group members and their affiliations.

**Table 1:** Advisory board and affiliations

Advisory Board Member	Affiliation
Robert J. Alvey	Public Information Officer Bioterrorism Preparedness Team, Arkansas Department of Health
C. Kirk Avent, MD	Medical Director of Disease Control Jefferson County Department of Health, Birmingham, Alabama
Steven M. Becker, PhD	Associate Professor University of Alabama at Birmingham, School of Public Health, Birmingham, Alabama; Member, Scientific Committee 46-14, NCRP
Eric Braun	Vice President for Convergence and corporate news director Raycom Media, Inc., Montgomery, Alabama
Jerdan Bullard	Executive Director, Alabama Broadcasters Association, Birmingham, Alabama
Joy Carter	Media Relations, University of Alabama at Birmingham, Birmingham, Alabama
Vincent T. Covello, PhD	Director, Center for Risk Communication, New York, New York
Sharon Dunwoody, PhD	Evjue-Bascom Professor and Director University of Wisconsin, School of Journalism and Mass Communication, Madison, Wisconsin
Jamey Durham	Risk Communication Specialist, Alabama Department of Public Health, Montgomery, Alabama
David R. Franz, DVM, PhD	Chief Biological Scientist, Midwest Research Institute, Kansas City, Kansas; Director, National Agricultural Biosecurity Center, Kansas State University
Sharon Friedman, PhD	Professor of Journalism and Communication; Director of the Science and Environmental Writing Program; Lehigh University, Lehigh, Pennsylvania
L. Sam Hansen, EMT-P	Battalion Chief, Vestavia Hills Fire Department, Vestavia Hills, Alabama; Instructor, Texas A&M University National Emergency Rescue esponse Training Center
Captain David K. Hartin	Public Information Officer, Tuscaloosa Police Department and Tuscaloosa County Emergency Management Agency, Tuscaloosa, Alabama
Robert J. Howard, MS, MPH	Consultant. Former press officer and Director of Strategic Communication, National Centers for Infectious disease, Centers for Disease Control and Prevention, Duluth, Georgia
Corporal Don Kelly	Baton Rouge Police Department, Office of the Chief of Police, Media Relations Division; Past President, National Information Officers Association
Donald Koss	Instructor, Mississippi Fire Academy, Pearl, Mississippi; Former Public Information Officer, Hattiesburg (MS) Fire Department
Deborah Potter	Executive Director, Radio-Television News Directors Foundation
Scott C. Ratzan, MD, MPA	Editor, Journal of Health Communication; Vice President, Government Affairs, Johnson & Johnson Europe (Belgium)
Thomas E. Terndrup, MD	Professor and Chair, Department of Emergency Medicine; Director, Center for Emergency Care and Disaster Preparedness, University of Alabama at Birmingham, Birmingham, Alabama
Laura Tutor	Metro Editor, Anniston Star, Anniston, Alabama
Edward Van Horne	Executive Director, Southern Newspapers Publishers Association, Atlanta, Georgia
Chris Waddle	Director, Ayers Family Institute for Community Journalism; Vice President for News, Consolidated Publishing; Publisher, Anniston Star, Anniston, Alabama
Ann Wright	Public Information Officer, Arkansas Department of Health; Regional Representative (Region VI), National Public Health Information Coalition

The advisory group members represent several distinct constituencies, including constituencies that sometimes conflict during emergencies related to terrorism and natural disasters – most notably, journalists and public information officers. Views across constituencies were compared, to identify areas of agreement and disagreement regarding pressing problems and likely solutions. Accordingly, interviews occasionally segregated the advisors, speaking with only one particular constituency (e.g., experts in terrorism). During the January 29, 2004, meeting of the advisory group, separate "break-out" discussions were held with advisors who represent two particular constituencies: journalists and PIOs. The group was then reconvened as a whole to discussed points and issues that had emerged from the break-out sessions.

## Results

This section addressed what advisors said were the major problems to be addressed in any effort to improve media coverage and public understanding of bioterrorism and emerging infectious diseases.

### Problems related to news content

Advisors frequently lodged two complaints regarding media coverage of health-related emergencies. First, they complain that stories are too often hectic and breathless, that the need for journalists to file stories frequently and quickly leads to stories that focus on details and events that experts deem to be irrelevant, unimportant, unhelpful, and sometimes inaccurate. Second, they complained that stories are too often shallow and do not provide much contextual information, focusing instead on whatever details may be new, providing little attention to how these details may be related to a comprehensive understanding of the situation.

### Problems related to journalists' work practices

Advisors complain that too many journalists' stories fail to reflect the complexities of health-related emergencies. Accordingly, journalists may find it difficult to create stories about terrorism or natural disasters in which complexity is highlighted rather than minimized. Another limitation, according to journalism advisors, is the decreasing commitment by news organizations to staff development and training in these content areas.

Advisors also perceive that journalists cannot tolerate uncertainty. They say journalists may mistake experts' typically cautious and hedging language about health-related emergencies as evidence of stonewalling or incompetence, and that journalists too eagerly seek sources – sometimes less reputable sources – who will speak with relatively less caution.

Advisors also perceive that journalists are unreasonably impatient. Few of the professional groups with which journalists interact are similarly subject to tight, unyielding deadlines. To PIOs and terrorism experts among the advisors, it seems that journalists too frequently make unreasonable demands, looking for instant access to information that does not yet exist or demanding that PIOs and experts offer statements on issues that PIOs and experts feel unprepared to address. Confrontation also derives from what PIO's perceive as distrust on the part of journalists. They say many journalists make little effort to hide their distrust, and many openly profess to be adversaries of PIO's, citing their role as "watchdogs" on government institutions.

### Problems related to PIO work practices

Journalism advisors have several complaints about the processes and practices of PIO's during health-related emergencies. Several say PIO's commonly err on the side of withholding access to information and experts, especially in situations in which the PIO fears that access will result in a story that hinders rather than helps emergency response. When this happens, they say, PIO's become bottlenecks rather than useful gatekeepers, facilitators, and translators.

The journalism advisors complain that PIO's sometimes lack the authority to provide access to information and experts. PIO's may not be permitted by their superiors to provide access to the information and experts that journalists seek, and they may not know what information and experts are [3] available or [4] appropriate and helpful. Journalism advisors also complain that PIO's sometimes overlook obvious opportunities to facilitate information dissemination and access to experts. For example, online resources could be especially helpful in emergency situations in which PIO's are deluged with requests for information.

Finally, broadcast journalists complain that PIO's too often perform poorly on camera and in interviews. This problem is more common at state and local agencies than at national agencies, where PIO's are more likely to receive training in these skills. PIO's may underestimate journalists' needs for PIO's who can themselves become a useful, visible part of a story, rather than merely serve as an access point for information.

It should be emphasized that the PIO advisors believe many of these problems are being addressed. They note that PIO's at almost every state public health agency have participated in training that aims to facilitate interaction with journalists. Since 9/11, the advisors note, PIO's have become far better prepared to support journalists who will cover terrorism and other disasters.

### Problems with both journalists and PIO's

The advisors complain that in responding to health-related emergencies both journalists and PIO's frequently do not understand the likely impact of news coverage. They may be overly concerned with negative outcomes that are in fact unlikely, such as public panic – so concerned that they withhold helpful information. The PIO advisors say PIO's have learned that media reports are unlikely to cause panic and that PIO's must never withhold information. But in general, the advisors agree that journalists and PIO's still have much to learn about the impact of media coverage.

Both journalists and PIO's profess to be mystified or dismayed by what they believe readers and viewers want to know about health-related emergencies. They worry about their ability to convey information about likely risks and threats in a way that increases audience concern when concern is appropriate but that does not cause audiences to discount media reports after long periods in which danger is covered only as a possibility. In addition, neither journalists nor PIO's perform well in terms of addressing the information needs of special populations during health-related emergencies.

Interaction between journalists and PIO's is another key issue. The two fields are professionally segregated and practitioners are typically trained in separate degree programs in colleges and universities. This segregation has roots in sensible and important efforts to maintain professional standards, but it may hinder their interaction in response to health-related emergencies. Journalists and PIO's are often on good terms, but they are in fragile, fleeting relationships. Advisors say journalists can readily name a PIO whom they trust as an ally in creating responsible coverage of health-related emergencies. Similarly, most PIO's can identify a journalist whom they consider a partner in responding effectively to health-related emergencies. Unfortunately such relationships appear to be exceptional and short-lived due largely to frequent personnel changes. But according to the advisors, journalists and PIO's believe it is necessary to build close relationships if they are to respond effectively to health-related emergencies.

### Organizational issues

For decades, television, radio, newspaper, and magazines have competed zealously against one another. Cooperation became somewhat more common in the 1990s as media mergers and acquisitions escalated (bolstered by media deregulation), bringing together one-time foes who are now expected to realize "synergies" through cross-media cooperation. Technological convergence now facilitates cooperation. According to the advisors, journalists who cover health-related emergencies seem increasingly eager to cooperate with colleagues across media; though to date there have been few efforts. However, the advisors indicated that journalists learned via the events of September 11, 2001, that media cross-media collaboration is necessary if media are to respond effectively to health-related emergencies. Advisors say more cooperation with radio could reap considerable benefits, as radio's ubiquity and portability may make it the most effective medium for disseminating news during emergencies. However, as radio station groups become bigger, fewer in number, and centrally rather than locally programmed, they employ fewer community journalists. As a result, unlike other media, radio is becoming less able to respond to health-related emergencies.

### Problems related to emergency preparedness

Journalists have yet to pursue coordinated, programmatic efforts to prepare for emergency response. The advisors identify three key problems in this area. One is that most media outlets lack routinely-updated, viable emergency response plans, and in cases where outlets have viable plans, journalists seldom practice these plans. A second problem is that too many of the (few) media outlets that are developing emergency response plans exclude PIO's in this planning. At the same time, journalists are not frequently invited by public health and emergency management agencies to participate in state and local preparedness drills and simulations. This failure to explicitly include both journalists and PIO's in formal emergency planning may doom these plans to failure. The third problem is that journalists could do a better job of communicating emergency response plans that have been developed by government agencies. The advisors agree that government agencies and news organizations should work together to publicize emergency response plans.

### Problems related to resource development, availability, and dissemination

The advisors do not believe that journalists and PIO's are without resources to support them as early responders to health-related emergencies. However, they deem this a key problem area, for three reasons. First, such resources are few, scattered and fugitive. Most journalists and PIO's can identify a resource (e.g., a simulation, a workshop, a course, a book) that they have found helpful. However, few journalists and PIO's nominate the same resource. Moreover, journalists and PIO's are frequently unaware of resources that others have found helpful unless and until a colleague recommends a resource. Second, resources are needlessly duplicated. There is a dearth of cross-organizational cooperation in developing and sharing resources. Third, new technologies such as online media have not yet been harnessed to effectively develop and disseminate resources [19]. The barriers to improve journalist and public health information officer's communication and their solutions are summarized in Table 2.

**Table 2:** Barriers and solutions to improve journalist and public information officers (PIO) communication in health-related emergencies

Barrier	Solution
1. Media stories are too often hectic and do not provide much contextual information	1. Develop case-based learning opportunities to illustrate best practices
2. Media stories fail to reflect the complexities of health-related emergencies	2. Invite journalist to participate in emergency and disaster drills and exercises
3. Media personnel lack access to information and experts	3. Facilitate contact and relationship-building between journalists and PIO's at the local level
4. Lack of training to convey information about likely risks and threats in appropriate manner to the public	4. Provide experiential learning opportunities for both journalist and PIOs
5. Journalist lack of cooperation for information dissemination with colleagues across other media types	5. Use the Internet and other mobile technologies to disseminate information
6. Lack of government agencies and media organizations in promoting and publicizing emergency response plans together	6. Coordinate with public health and other government agencies in developing emergency response plans.
7. Lack of consensus between journalists and PIOs related to resource development, availability, and dissemination for responders	7. Bring together the various entities that are developing resources and publish information through trade publications and professional meetings

#### Recommendations

Below we offer recommended solutions to these and other problems identified. We are keen to make recommendations that are feasible in terms of costs and practical in terms of the professional and organizational realities in which journalists and PIO's work. To support journalists and PIO's as first responders to terrorism and other health-related emergencies, it will be useful to:

1. Provide experiential learning. Even the advisors who lead university degree programs in journalism are quick to join other advisors in forecasting that journalists and PIO's can benefit from experiential learning (as opposed to traditional, formal education).

2. Keep it affordable (cheap, even). Journalists and PIO's work under organizational constraints and do not enjoy large budgets for professional development.

3. Keep it brief. Journalists and PIO's find it difficult to devote more than two consecutive days wholly to learning opportunities. Even two days may be too much. One-day and one-half-day programs should be considered when feasible.

4. Go to them. Journalists and PIO's have professional orientations and long traditions of attendance at annual meetings of national, regional, and state organizations. These meetings provide an ideal opportunity for resource dissemination.

5. Keep it nearby. Other things being equal, journalists and PIO's are better able to participate in local and regional events than in national events.

6. Keep it "in-house." Due to clashing professional norms, as well as a preference for known practices and personnel, journalists and PIO's will be most responsive to resources developed and disseminated by their own professional and trade organizations.

7. Work together. Support journalists in pursuing their growing interests in, and growing need for, collaboration across media. Persuade journalists and PIO's to demand resources designed specifically to support journalist-PIO interactions.

8. Make local connections. Facilitate contact and relationship-building between journalists and PIO's at the local level, helping the personnel who will interact in emergencies anticipate and respect the professional needs of one another.

9. Use scenarios and cases. The rapidly growing interest in and use of, scenario- and case-based learning should be embraced as an especially effective and seemingly popular form of experiential learning. Develop innovations in scenario-based learning that invite journalists to learn literally to work as PIO's, and vice-versa.

10. Participate in drills and exercises. Gain standing for journalists and PIO's to participate routinely in disaster response drills and exercises. Assess the effectiveness of drills in part based on how well journalists and PIO's perform and how well other agencies perform in interacting with journalists and PIO's.

11. Use the Internet and other mobile technologies to develop and disseminate resources.

12. Take advantage of existing programs and publications. Make effective use of trade publications and professional meetings to provide information about available resources

13. Translate and disseminate the work of experts in health and risk communication. There is an already large and steadily growing body of research in health and risk communication that addresses several of the problems identified above, especially the problems related to audience interest, attention and understanding and the impact of news coverage. This research is underutilized by journalists and PIO's who shape media coverage of terrorism and other disasters but who have an inadequate understanding of the nature and needs of audiences.

14. Get on the same page. Bring together the various entities that are developing resources, or at least ask them to better coordinate efforts and more effectively disseminate resources. Establish more effective channels of communication not just across resource providers but also between resource providers and the journalists and PIO's they aim to support.

15. Plan. It is crucial that news organizations develop emergency response plans. Moreover, this planning should be done in coordination with public health and other government agencies. This planning should be informed by the growing literature on emergency planning [20].

## Discussion and conclusion

The recommendations listed offer no guarantee of improvement. Much will depend on the specific details of resources developed. Effective solutions will require careful planning, ongoing evaluation and a continuing realization of the larger organizational, professional and social factors that shape the problems being addressed. Many of the recommendations are consistent with recommendations emerging from recent conferences [21] and publications [10]. With this manuscript we contribute to the process of building consensus regarding pressing problems and likely solutions.

It is clear that journalists and public health spokespersons play crucial roles in shaping public response to terrorism and other disasters [2]. Journalists and PIO's are professionals who frequently interact in covering health-related emergencies. Unfortunately, they are often inadequately prepared for this interaction, and they rarely coordinate efforts to become better prepared. As mentioned, there is a host of organizational, legal and professional constraints that play a role in this inadequacy. Nevertheless, legislative bodies, public health agencies, and media organizations should deem it a high priority to improve dissemination of information regarding terrorism and other disasters. Reliable and responsible news coverage should be seen as integral part of preparedness.

## Competing interests

The author(s) declare that they have no competing interests.

## Authors' contributions

WL conceived of the study and participated in the design and coordination and helped to draft the manuscript. WE conceived of the study and participated in the design and coordination and helped to draft the manuscript. KKG participated in the design and coordinated activities and helped to draft the manuscript. JAR participated in the design and coordinated activities and helped to draft the manuscript. PMG coordinated activities related to the study and helped to draft the manuscript. LCM coordinated activities related to the study and helped to draft the manuscript. MA coordinated activities related to the study and helped to draft the manuscript. All authors read and approved the final manuscript.

## Pre-publication history

The pre-publication history for this paper can be accessed here:


